# Development of a refined experimental mouse model of myasthenia gravis with anti-acetylcholine receptor antibodies

**DOI:** 10.3389/fimmu.2025.1521382

**Published:** 2025-03-31

**Authors:** Axel You, Léa S. Lippens, Odessa-Maud Fayet, Solène Maillard, Laureline Betemps, Antony Grondin, Jean-Thomas Vilquin, Nadine Dragin, Rozen Le Panse

**Affiliations:** Sorbonne University, Institut National de la Santé et de la Recherche Médicale (INSERM), Association Institute of Myology, Center of Research in Myology, UMRS 974, Paris, France

**Keywords:** autoimmunity, myasthenia gravis, experimental model, refinement, adjuvant, poly(I:C), LPS

## Abstract

Myasthenia gravis (MG) is an autoimmune disorder primarily caused by autoantibodies that target the acetylcholine receptor (AChR) at the neuromuscular junction (NMJ). The classical experimental autoimmune myasthenia gravis (C-EAMG) mouse model has long been used by immunizing mice with acetylcholine receptor from Torpedo fish (T-AChR), combined with complete Freund’s adjuvant (CFA). This mixture is administered via subcutaneous injections into the hind footpads and back, but CFA often causes strong inflammatory reactions, including lesions at the injection sites. Our objective was to develop a new EAMG model (N-EAMG) that is more compliant with animal welfare. C57Bl/6 mice were immunized twice weekly by intraperitoneal (i.p.) injection of T-AChR with a poly(I:C) and lipopolysaccharide (LPS) adjuvant mix. Control mice were injected with either physiological saline or the adjuvant mix alone. Various doses and injection schedules were tested, and the new model was compared with C-EAMG. Clinical symptoms were scored, antibody subtypes against T-AChR and mouse AChR were measured, and NMJ morphology and functionality were evaluated. We demonstrate that the N-EAMG model is at least as effective as the C-EAMG model. Moreover, similar to the C-EAMG model, the N-EAMG model is characterized by the production of T-AChR and m-AChR antibodies. This model also exhibited alterations in transmission at the NMJ due to antibody attack, resulting in a decrease in AChR surface area and increased AChR fragmentation. Symptoms were similar in both models but appeared more rapidly in the N-EAMG model. In addition, investigating the sensitization mechanism, we showed that i.p. injections of T-AChR with the poly(I:C)/LPS adjuvant mix, led to the recruitment in monocytes and changes in the two peritoneal macrophage subpopulations that were able to phagocytose T-AChR. These observations suggest that macrophage subtypes, albeit with varying efficiency, present the T-AChR to immune cells, leading to a specific immune response and the development of anti-AChR antibodies. In conclusion, our results demonstrate that this novel EAMG model is as effective as the C-EAMG model and offers several advantages. In particular, this model is more suitable for animal welfare and can replace the classical model in preclinical and fundamental research.

## Introduction

Myasthenia Gravis (MG) is an autoimmune neuromuscular disorder characterized by defective transmission of nerve impulses to muscles. Patients experience fluctuating muscle weakness that worsens with activity and improves at rest. MG is caused by autoantibodies against components of the neuromuscular junction (NMJ), primarily acetylcholine receptors (AChR). AChR-MG is associated with thymic changes, such as B-cell infiltration associated with lymphofollicular hyperplasia or thymoma (1).

Experimental mouse models of the AChR-MG (EAMG) have been used for several years. The susceptibility to MG varies depending on the mouse strain, and strains carrying the H-2b haplotype are more susceptible (2). Currently, C57Bl/6 mouse strain is the conventional wild-type mouse strain used in most studies (3). EAMG was induced by immunizing mice with a mixture of AChR purified from Torpedo fish (T-AChR) and complete Freund adjuvant (CFA). CFA corresponds to incomplete Freund adjuvant (IFA, paraffin oil) with the addition of heat-killed Mycobacterium tuberculosis (MTB). The first immunization was followed by one or two boosters at approximately 4-week intervals (3). In this model, mice develop antibodies against the T-AChR that cross-react with the AChR in the NMJ. However, this model has several limitations. 1) Mice need one or two injection boosts because symptoms can take 2-3 months to appear (3). 2) Only 50–70% of animals develop MG symptoms, whose severity can vary from one animal to another (3). 3) For initial immunization, the mice are injected at different sites, including the footpads. CFA leads to strong inflammation of the footpads, which can be painful for animals and alter the evaluation of clinical signs. Granulomatous lesions can develop at various injection sites (4). Therefore, a refined animal welfare protocol is needed. 4) Even though the EAMG model is relevant to study muscle weakness caused by AChR antibody attack, it does not completely recapitulate human disease, as the thymus is not inflammatory and is not characterized by B-cell recruitment associated with lymphofollicular hyperplasia (5). However, to date, no other model has replaced the EAMG model (hereafter referred to as C-EAMG for the classical-EAMG model).

LPS injections have already been used in the EAMG mouse model to try to substitute MTB in CFA as both LPS and MTB activate toll-like receptor (TLR)4. C57BL/6 mice were immunized by several subcutaneous (s.c.) injections in shoulders and footpads. They develop clinical signs similar to those observed with the AChR/CFA immunization (6). Using polyinosine polycytidylic acid (poly(I:C)), a synthetic analog of double-stranded RNA, we demonstrated that repeated intraperitoneal (i.p.) injections in C57BL/6 mice for 6–8 weeks induce an anti-AChR autoimmune response characterized by the production of anti-AChR antibodies, specific proliferation of B cells, and MG clinical signs. This induction is associated with transient thymic changes characterized by thymic inflammation, the overexpression of α-AChR subunit (the primary immunogenic subunit) and B-cell recruitment and proliferation (7, 8). We also showed that LPS potentiated poly(I:C) effects. Using a combination of poly(I:C) and LPS, we observed that some thymic changes were maintained after 6 weeks. In addition, i.p. injections of poly(I:C) during the course of the C-EAMG model potentiate the development of MG symptoms (9).

Here, gathering our knowledge on the effects of poly(I:C) and LPS injections, our objective was to develop a new EAMG model (N-EAMG). We investigated the effect of i.p. injections of T-AChR using a poly(I:C) and LPS adjuvant mix. We demonstrated that the N-EAMG model is as efficient as the C-EAMG model in inducing anti-AChR antibodies, impacting the structure and function of the NMJ, and triggering MG-like symptoms. These results suggest that the N-EAMG model is a more refined alternative to the classical-EAMG model.

## Methods

### Animals and T-AChR preparation

For this study, 6-week-old C57BL/6 female mice were purchased from Janvier Labs (Saint-Berthevin, France) and housed for 1–2 weeks before the experiments in a specific pathogen-free animal care facility (UMS28, Sorbonne University, Paris, France). Throughout the experiments, mice were regularly monitored for signs of muscle weakness, and mice that were too weak, as defined by the protocol, were euthanized. This study was approved by the local Ethics Committee (approval no. 29942). The extraction of Torpedo Californica AChR (T-AChR) was led as previously described (9).

### Classical experimental autoimmune myasthenia gravis

The T-AChR was emulsified with an equal volume of CFA (F5881, Thermo Fisher Scientific, Villebon-sur-Yvette, France) supplemented with heat-inactivated MBT (10 mg/mL, H37RA, BD Difco, Villepinte, France). Mice were s.c. injected (200 µL containing 30 µg T-AChR/mouse) at several sites (hind footpads, tail base, and in the back). Control mice were injected with a CFA emulsion lacking the T-AChR. After 3–4 weeks, the mice were re-immunized with T-AChR emulsified in CFA and administered solely at the tail base and back. To prevent potential pain associated with the injection into the footpads, the mice received a s.c. injection of buprenorphine prior to the procedure, and buprenorphine was added to their drinking water during the 1st week.

Depending on the experiment, mice were euthanized either 2 weeks after the last immunization or later in the case of time-course experiments for the assessment of immunopathological parameters.

### New experimental autoimmune myasthenia gravis

N-EAMG was induced by i.p. injection (twice weekly) of an adjuvant mix composed of poly(I:C) (200 µg/mouse) and lipopolysaccharide (LPS, 10 µg/mouse) containing T-AChR at different doses, depending on the experiment. The mice were injected for 4–6 weeks. Control mice were similarly injected with poly(I:C)/LPS mix or physiological water. Analgesics were not required in this model. We observed only slight feverishness in the mice on the day after injection.

Depending on the experiment, mice were euthanized either 2 weeks after the last immunization or later in the case of time-course experiments for the assessment of immunopathological parameters.

### Clinical evaluation of mice

Different assessments were performed to evaluate the clinical state and to calculate a global clinical score for each animal (**Supplementary Figure S1**). The mice were weighed once week. Muscle strength was analyzed by measuring forelimb strength using a grip strength apparatus (Bio-GS3, Bioseb, France) after a 5-min run on a treadmill (14cm/s). An inverted grid test was performed by gently dragging the mouse across the top grid of the cage 10 times. The grid was then rotated, and the time at which each mouse fell off was recorded. During all tests, the behaviors of the mice, such as posture and gait, were also recorded. A global clinical score ranging 0–9 was assigned based on weight, muscle strength, and a combined score for the inverted grid test and behavior. Each component was graded on a scale of 0–3, as described by Robinet et al. with minor modifications (**Supplementary Figure S1**) (9). The mice were considered sick when they reached a global clinical score of 2. Mice with a global clinical score of 9 were euthanized.

### Electromyography

Compound muscle action potential (CMAP) was measured in the hind limbs using s.c. placed electrodes as previously described (10). The sciatic nerve was stimulated using 10 stimuli at 1 Hz, 5 Hz, 10 Hz, 20 Hz, 30 Hz, and 40 Hertz (Hz). These measurements were performed on the muscle function assessment platform of UMS28 (Animal Facility, Sorbonne University). Peak-to-peak amplitudes were analyzed using the LabChart Reader software to determine the amplitude (mV) from the maximum negative to positive peaks of the biphasic wave.

### Immunostaining of muscle isolated fibers

Immunofluorescence labeling was performed on the isolated muscle fibers as previously described with minor modifications (10). The tibialis anterior muscle sections were fixed with a 4% paraformaldehyde (PFA) solution in phosphate-buffered saline (PBS) for 1 h at room temperature. Muscle fibers were dissected, rinsed in PBS for 15 min, and incubated in 0.1 M glycine in PBS at 4°C overnight. Next, the fibers were washed three times with PBS for 10 min, permeabilized and blocked with a 4 h incubation at room temperature in a solution containing 4% of bovine serum albumin (BSA), 5% goat serum, and 0.5% Triton X-100 in PBS.

To stain the presynaptic terminals, the fibers were incubated overnight at 4°C with primary mouse antibodies against synaptic vesicle glycoprotein 2A (SV2) (DSHB, University of Iowa, USA) and neurofilament (NF) (2H3-C, DSHB) diluted 1:500 in a blocking solution. Next, the fibers were washed hourly in a 0.1% Triton X-100 solution throughout the day and stained with AF488-conjugated goat anti-mouse antibody (1:500, A21121, ThermoFisher Scientific). To detect AChR on the postsynaptic area, tetramethyl rhodamine-conjugated α-bungarotoxin (1:500, T1175, ThermoFisher Scientific) was added together with the secondary antibody for an overnight incubation at 4°C in the dark with gentle stirring.

After several hourly washes in 0.1% Triton X-100 in PBS throughout the day, the muscle fibers were mounted using VECTASHIELD mounting medium (H-1000, Vector Laboratories, Eurobio scientific, Les Ulis, France) (10). Image were acquired using a Zeiss Axio Imager M2 microscope equipped with an ApoTome 2 module, using a Plan-Apochromat 63x/1.3 NA oil DIC objective to obtain z-stack sets of images capture at multiple focal planes separated by regular intervals. Next, Images were analyzed and presented as maximum intensity projections derived from z-stacks. Image analysis was conducted using the ImageJ software.

### Enzyme-linked immunosorbent assay for anti-T-AChR and m-AChR antibodies

For ELISA, 96-well plates were coated with 0.5 µg/mL of T-AChR or m-AChR peptide (#AP73672, Signalway Antibody/Biovalley, Nanterre, France) diluted in 10 mM NaHCO_3_ buffer, pH 9.6, overnight at 4°C. Wells were blocked with PBS containing 10% fetal calf serum for 150 min at 37°C. Serum samples were diluted in PBS containing 0.2% BSA (1:200000 or 1:200 for T-AChR or m-AChR ELISA, respectively) and incubated at 37°C for 90 min. 100 µL of biotinylated rabbit anti-mouse immunoglobulin (Ig)G (1/1800, E0413, Dako, Courtaboeuf, France) or biotinylated anti-mouse IgG subtypes 1/250 were added for 90 min at 37°C (anti-IgG2b (553393) or anti-IgG1 (553441) from BD Biosciences, Le Pont de Claix, France). Samples were incubated 30 min with 100 µL of streptavidin–horseradish peroxidase (1:10000) (S911, ThermoFisher Scientific). Tetramethylbenzidine was used for color development, and the optical density was measured at 450 nm using a SPARK 10M microplate reader (TECAN Life Sciences, Grödig, Austria). Between each step, wells were washed four times with 200 µL of PBS 0.05% Tween 20.

### Detection of avidity of anti-AChR IgG antibodies

The relative avidity of the anti-AChR antibodies was determined based on the above ELISA method with T-AChR coating using a potassium thiocyanate (KSCN) elution step (11). Increasing concentrations of KSCN were added and incubated for 15 min at room temperature before incubation with biotinylated rabbit anti-mouse IgG. KSCN concentrations ranging from 0.25 to 4 M were used. Data were log-transformed to form a standard curve, and the relative affinity corresponded to the molarity of KSCN, resulting in 50% of the absorbance obtained in the absence of KSCN.

### Analysis of peritoneal phagocytic cells

Mice were injected with physiological water, the adjuvant mix composed of poly(I:C) (200 µg)/LPS (10 µg) with or without T-AChR. T-AChR was labeled with Texa-Red using LYNX Rapid Plus DyLight^®^488 Antibody Conjugation Kit (ThermoFisher Scientific). Next, the mice were euthanized, and peritoneal cells were harvested by injecting 10 mL of cold PBS containing 3% SVF into the cavity. Peritoneal cells were counted and stained with a LIVE/DEAD™ kit (LD34960D, ThermoFisher Scientific) and for 30 min at 4°C with the following antibodies: anti-CD19-APC (152409) from Biolegend (Amsterdam, The Netherlands), anti-CD3-APC (17-0032-82), anti-MHC Class II-FITC (11-5322-82), anti-F4/80 eFluor-450 (48-4801-82) from e-Biosciences (ThermoFisher Scientific), anti-CD11b PE-Cy7 (552850), anti-CD11c-Alexa Fluor 700 (560583), anti-Ly6C BV605 (563011) from BD Biosciences. The cells were washed with PBS before flow cytometric analysis on a Cytoflex S. Data were analyzed using CytExpert (Beckman Coulter).

### Statistical analysis

GraphPad Prism 9 was used for statistical analyses and graphic representations. For two-by-two comparisons, the non-parametric Mann-Whitney U test was used. To analyze the mouse susceptibility to EAMG, the comparisons among the different mouse groups were performed using two-way analysis of variance (ANOVA) with Tukey’s *post-hoc* test for multiple comparisons.

## Results

### Proof-of-concept: Effects of i.p. injections of T-AChR with a poly(I:C)/LPS adjuvant mix

To test if repeated i.p. injections of T-AChR could induce a robust immune response against T-AChR, we used an adjuvant mix of poly(I:C) and LPS (hereafter termed “PL”) containing 10 µg of T-AChR for the new EAMG model (N-EAMG). The mice were injected twice week for 6 weeks.

**Figure 1:**
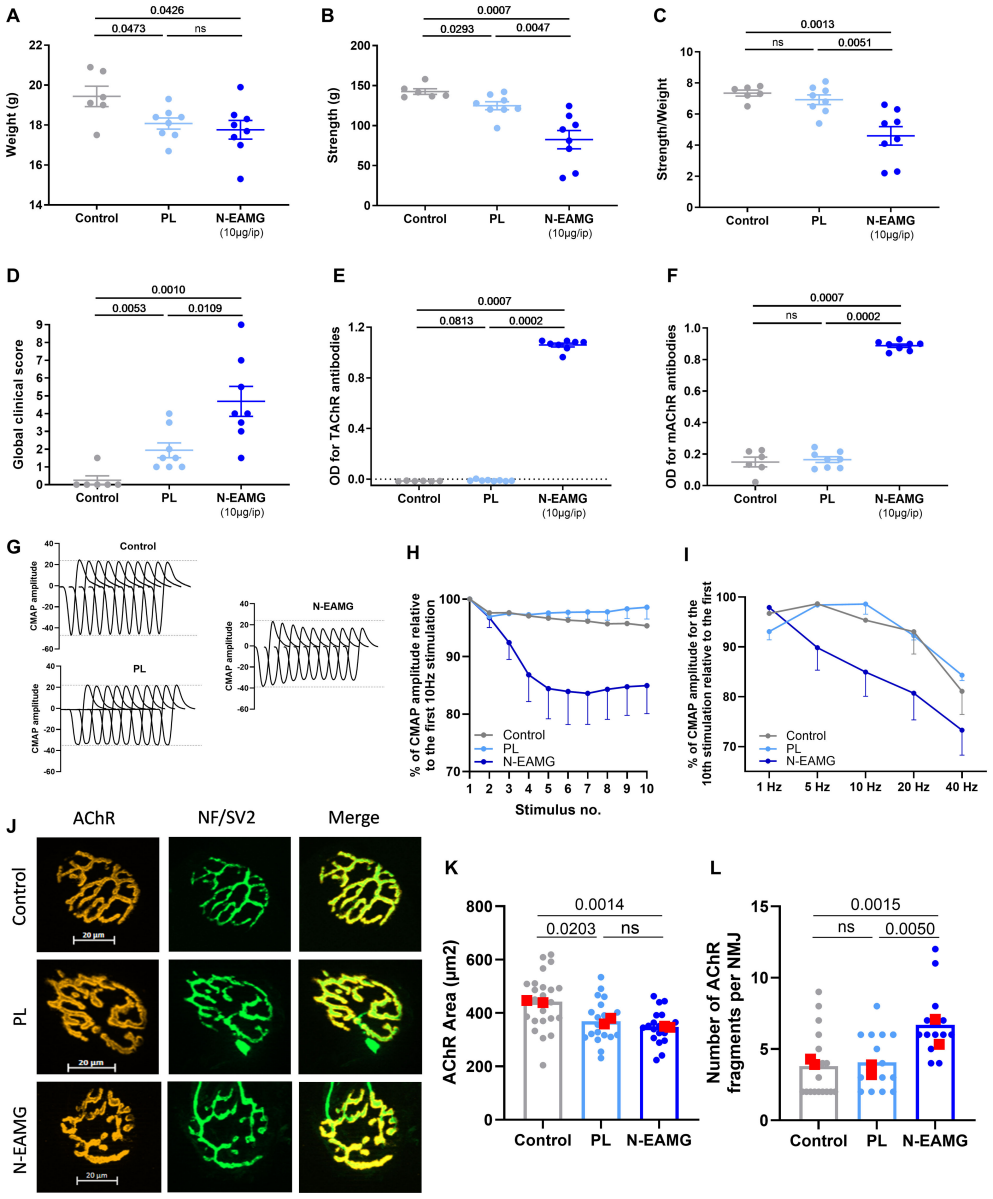
Induction of MG symptoms with i.p. injections of T-AChR: Initial proof-of-concept. C57BL/6 mice (n=6–8 per group) were i.p. injected twice weekly with physiological water (control), a poly(I:C)/LPS (PL) adjuvant mix, or a PL adjuvant mix with T-AChR 10 µg (N-EAMG). Clinical evaluations were performed after 6 weeks. **(A)** Mice were weighed. **(B)** Muscle strength was measured using a grip-test apparatus after exercise on a treadmill. **(C)** Muscle strength was normalized to mouse weight. **(D)** GCS for each mouse was calculated based on weight loss, grip test, and inverted grid test. **(E, F)** ELISA for anti-T-AChR and anti-m-ACHR antibodies detected with a global anti-IgG biotinylated antibody was performed on the serum collected at the end of the experiment. The integrity of neurotransmission and NMJ morphology were analyzed. Two mice with grip strength values close to the means of their respective groups were selected for these analyses. **(G)** CMAP was recorded in the TA muscles during a train of 10 sciatic nerve stimulations at 10 Hz. Representative traces of CMAP in control, PL, and N-EAMG mice. **(H)** Quantitative analysis of the CMAP amplitude after 10 Hz stimulation, expressed as a percentage of the first CMAP amplitude. **(I)** Quantitative analysis of the amplitude of the 10th CMAP after sciatic nerve stimulation at 1, 5, 10, 20, and 40 Hz. The data are expressed relative to the amplitude of the first CMAP of the train. **(J)** Representative images of isolated TA muscle fibers. Muscles were stained with α-bungarotoxin for detection of AChR (red) and antibodies directed against NF and SV2 (green) for the labeling of nerve terminals (scale bar = 20 µm). The number of AChR fragments was quantified from image stacks (control: 3 fragments, PL: 6 fragments, and N-EAMG: 7 fragments). **(K)** Quantitative analysis of the AChR-labeled area. **(L)** Number of AChR fragments isolated per NMJ. Each round point corresponds to one NMJ and each red square point corresponds to the mean value for each mouse. **(A–F, K, L)** P-values were assessed using the Mann-Whitney U test and indicated when p<0.1 or ns, not significant. **(H, I)** The data shown are the mean ± SEM (n=2; no statistical analyses were performed with such a small sample size).

We observed that the adjuvant mix itself induced a reduction in body weight and muscle strength, leading to a higher global clinical score (GCS) (**Figures 1A–D**). This effect has been described previously (9). However, the decrease in strength and increase in GCS score were stronger when T-ACHR was added to the adjuvant mix (**Figures 1A–D**).

Using ELISA, we measured high levels of T-AChR and m-AChR antibodies in the N-EAMG model (**Figures 1E, F**). A slight increase in T-AChR antibodies was induced through the PL adjuvant mix itself (**Figure 1E**), as previously described (9). This induction was more significant when serum samples were only diluted at 1/200: OD (mean ± standard error of the mean [SEM]) for control group = 0.049 ± 0.01 vs. the PL group = 0.176 ± 0.016, p= 0.0007) but less important compared to N-EAMG mice.

To set up the N-EAMG model, we also tested the effects of one i.p. injection of 20 µg of T-AChR vs. two i.p. injections weekly with 10 µg. After 6 weeks, we observed that one injection weekly was much less efficient in inducing T-AChR and m-AChR antibodies and MG symptoms compared to the N-EAMG model with two i.p. injections weekly (data not shown). Consequently, this approach has not yet been pursued.

To objectively prove that the observed muscle weakness was associated with a functional alteration at the NMJ level, we analyzed the integrity of neurotransmission by recording CMAP in the TA muscles during trains of 10 stimulations of the sciatic nerve at different frequencies and quantifying CMAP amplitudes. Peak-to-peak amplitude measurements showed that CMAP remained stable in control and PL mice subjected to 10 Hz stimulation, but clearly decreased in N-EAMG-treated mice (**Figures 1G, H**). We also analyzed the overall decrease at all tested frequencies by comparing the amplitude of the last CMAP of a train of stimuli with the first one. The decrease in CMAP amplitude was stronger in N-EAMG mice than in the control and PL mice (**Figure 1I**). Generally, a decrement of > 10% in the amplitude or area of the CMAP during a train of 3-Hz nerve impulses is considered evidence of impaired neuromuscular transmission (12). Consequently, our results demonstrated progressive neuromuscular weakness and fatigability in N-EAMG mice.

Next, we analyzed the morphological phenotypes of NMJ using immunofluorescence to identify functional defects. TA muscle fibers were stained with fluorescent α-bungarotoxin to label AChR, and with a mixture of antibodies against neurofilament (NF) and synaptic vesicle glycoprotein 2A (SV2) to label nerve branches and terminals, respectively. The NMJ of the control animals had a typical pretzel-like shape. However, increased fragmentation of the AChR network was observed in PL and N-EAMG mice (**Figure 1J**). Quantitative analysis revealed a significant decrease in the AChR area at the endplates (**Figure 1K**) and a significant increase in the number of AChR fragments (**Figure 1L**). These results clearly demonstrated the morphological alterations of NMJ in the N-EAMG model.

The results from this proof-of-concept analysis demonstrate that using an adjuvant other than CFA and an administration route other than footpads can induce MG-like muscle symptoms and defects in mice.

### Comparison of the N-EAMG model with the C-EAMG model

Three independent experiments were performed to optimize the T-AChR dose and compare the efficacies of the N-EAMG and C-EAMG models. Different batches and concentrations of T-AChR were used to induce the N-EAMG model: 5, 10, and 20 µg per injection.

**Figure 2:**
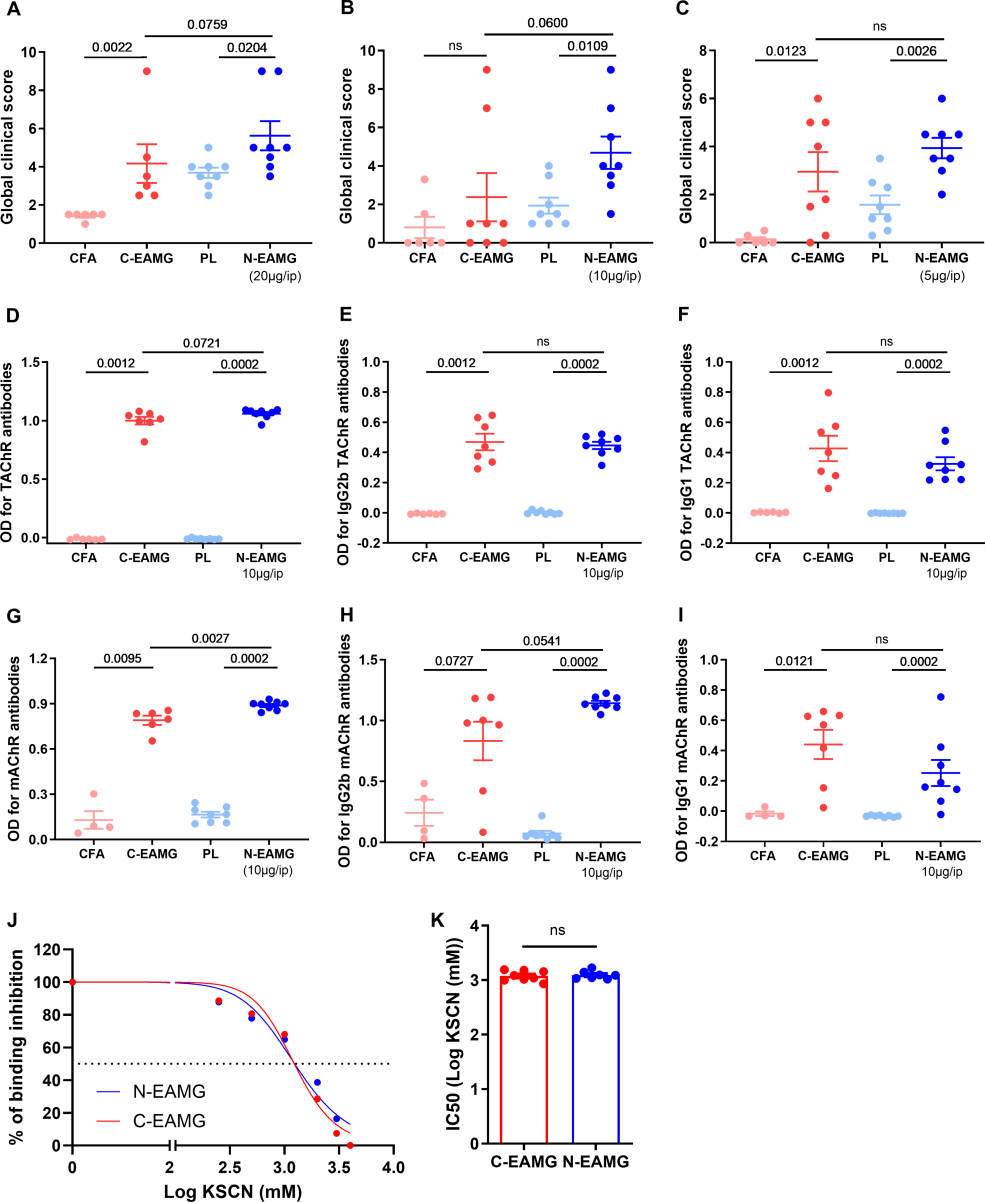
Comparison of the N-EAMG model to the C-EAMG model for MG symptoms. C57BL/6 mice (n=6–8 per group) were used in three experiments with different doses of T-AChR per injection. Mice were i.p. injected twice weekly with a poly(I:C)/LPS (PL) adjuvant mix or a LP adjuvant mix with T-AChR (N-EAMG) 20 **(A)**, 10 **(B)**, or 5 µg **(C)** per injection. For the C-EAMG model, mice were immunized with CFA/T-AChR (30 µg, C-EAMG) or just CFA on day 0 and boosted after 3–4 weeks. **(A–C)** Clinical evaluations were performed after 6 weeks, and GCS for each mouse was calculated considering weight loss, strength, and inverted grid test results. Details of the mouse weight and strength are shown in **Supplementary Figure 2**. **(D–F)** Anti-T-AChR antibodies were measured using ELISA and detected using anti-mouse IgG **(D)**, IgG2b **(E)**, and IgG1 **(F)** antibodies. **(G–I)** Anti-m-AChR antibodies were measured using ELISA and detected using anti-mouse IgG **(G)**, IgG2b **(H)**, and IgG1 **(I)** antibodies. **(J, K)** The relative affinity index of anti-T-AChR IgG antibodies was determined using KSCN thiocyanate, as detailed in the methods section. The binding inhibition curves represent the mean of the mice in each group **(J)**. The half-maximal concentration of KSCN (IC50) was required to inhibit the binding of anti-AChR antibodies in the C- and N-EAMG models **(K)**. P-values were assessed using the Mann-Whitney test to compare CFA and C-EAMG, PL and N-EAMG, C-EAMG and N-EAMG, and indicated when p<0.1 or ns, not significant.

For GCS, we observed that the N-EAMG model was more or more efficient (**Figures 2A–C**; **Supplementary Figures S2A–I**) than the C-EAMG model in inducing MG symptoms, regardless of the dose of T-AChR used. Selecting the intermediate dose of T-AChR (10 µg/mL) for the N-EAMG model, we analyzed the different subtypes of antibodies. The anti-T-AChR levels were similar regardless of the detection antibody used (anti-IgG, -IgG2b, or -IgG1) (**Figures 2D–F**), whereas the anti-m-AChR levels were always significantly higher in the N-EAMG model when detected with anti-IgG or -IgG2b but not anti-IgG1 antibodies (**Figures 2G–I**). The anti-T-AChR and m-AChR levels were also measured in the N-EAMG models obtained by injecting doses of 20 and 5 µg/mL of T-AChR. The results show that higher doses of T-AChR led to increased autoantibody levels in the N-EAMG model (**Supplementary Figures S3A–D**). To characterize the properties of anti-T-AChR antibodies in the C-EAMG and N-EAMG (10 µg/mL) models, we compared the antibody avidity but did not detect any difference between the two models (**Figures 2J, K**).

Although the mice used in the C-EAMG model often displayed swollen footpads that could alter clinical measures, this was not the case for the mice in the N-EAMG model. In addition, some mice in the C-EAMG model developed itching patches and skin lesions.

These results show that the N-EAMG model is as efficient as the C-EAMG model in inducing MG symptoms. In addition, this model is reproducible, as it was developed with different T-AChR batches and conducted by various experimenters.

### Longitudinal kinetic assessment of the N-EAMG and C-EAMG models

**Figure 3:**
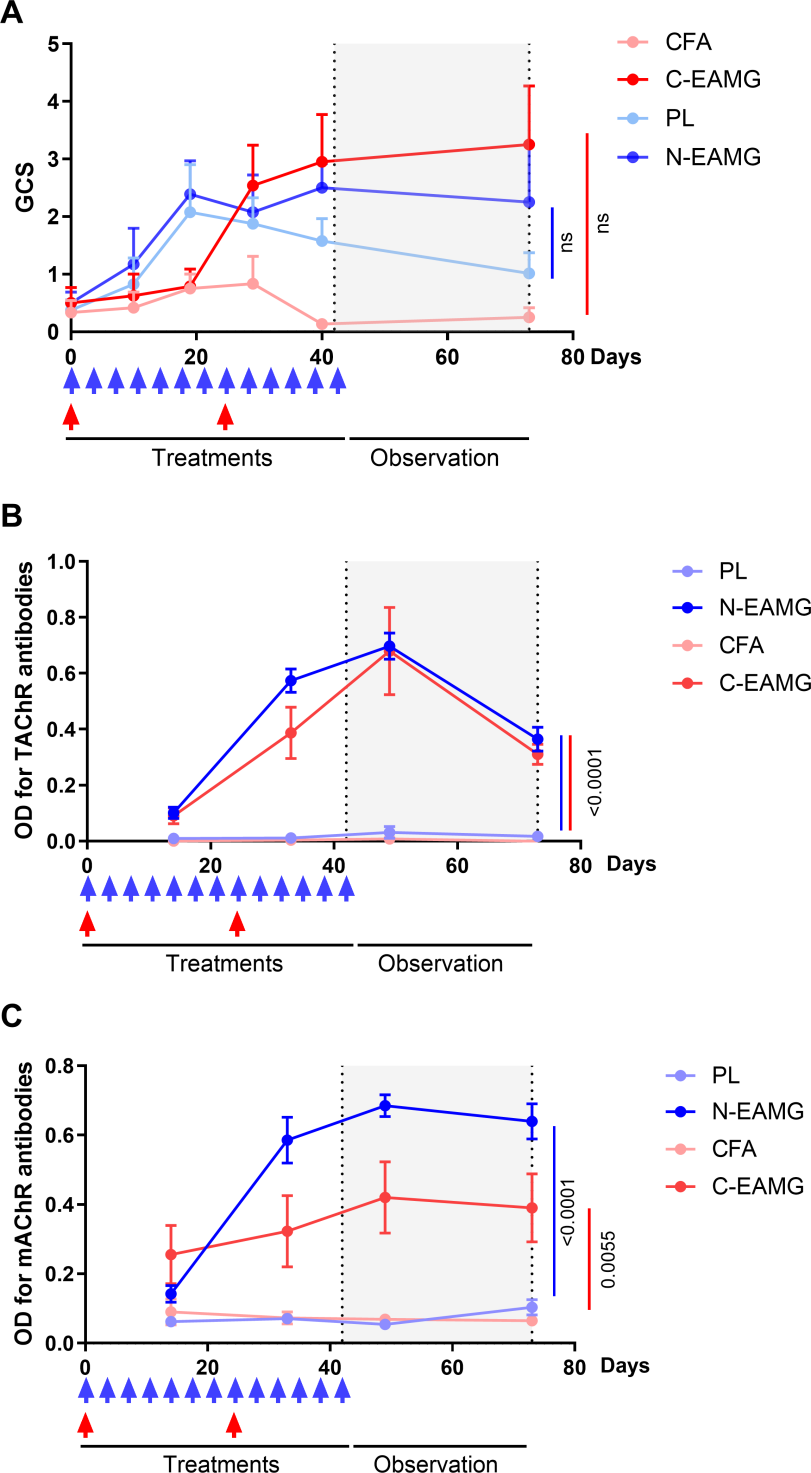
Comparison of long-lasting effects in the N-EAMG and C-EAMG models. C57BL/6 mice (n=6-8 per group) were used in the N-EAMG and C-EAMG models. For the N-EAMG model, mice were i.p. injected twice weekly with a Poly(I:C)/LPS (PL) adjuvant mix or a PL adjuvant mix with T-AChR 10 μg (N-EAMG). Mice were injected for 6 weeks, then injections were stopped and mice were observed up to 10 weeks. For the C-EAMG model, mice were immunized with CFA/T-AChR (30µg, C-EAMG) or just CFA at day 0 and boosted after 22 days. After the boost, mice were observed up to 10 weeks. **(A)** Clinical evaluations were performed regularly and the global clinical score (GCS) for each mouse was calculated considering weight loss, strength, and inverted grid test results **(B, C)**. Anti-T-AChR **(B)** and m-AChR **(C)** antibodies were measured using ELISA and detected using anti-mouse IgG. **(A-C)** Two-way ANOVA with Tukey post-hoc tests were performed to compare CFA and C-EAMG or PL and N-EAMG. p values were indicated when p<0.05.

Next, we compared the persistence of symptoms in the C-EAMG and N-EAMG models. For the C-EAMG model, the mice were immunized on day 0 and boosted 24 d later. For the N-EAMG model, mice were injected twice weekly with 10 µg of T-AChR and the poly(I:C)/LPS mix for 6 weeks and left untreated for 4 weeks thereafter. During the period of induction, GCS showed that symptoms appeared more rapidly in the N-EAMG model (**Figure 3A**) and reached a similar level after the boost in the C-EAMG model. Symptoms persisted in both models for up to 10 weeks (**Figures 3A, B**). Anti-T-AChR antibody titers were similar in both models (**Figure 3B**), whereas anti-m-AChR antibody titers were higher in the N-EAMG model (**Figure 3C**). Altogether, these results showed that MG symptoms and AChR antibodies appeared earlier in the N-EAMG model and persisted similarly after the discontinuation of injections, compared to the C-EAMG model.

**Figure 4:**
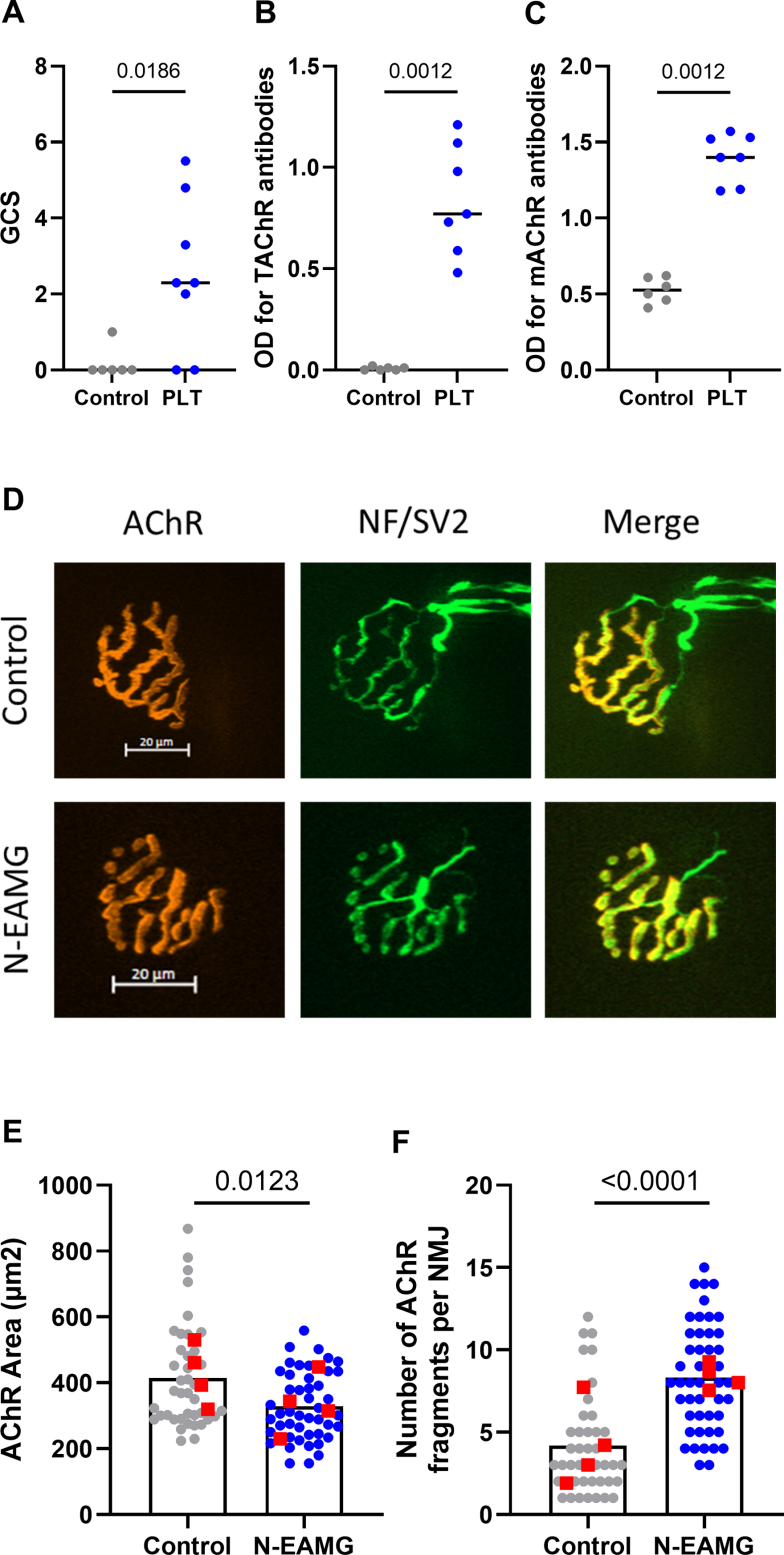
Long-lasting effects affecting the NMJ in the N-EAMG model. C57BL/6 mice (n=6–8 per group) were i.p. injected twice weekly with physiological water (control) or the poly(I:C)/LPS mix with 10 µg T-AChR per injection. The mice were injected for 4 weeks, the injections were stopped, and the mice were analyzed after 4 weeks. **(A)** Clinical evaluations were performed regularly, and GCS for each mouse was calculated based on weight loss, grip test, and inverted grid test. **(B, C)** Anti-T-AChR **(B)** and anti-m-AChR **(C)** antibodies were measured using ELISA and detected using anti-mouse IgG. **(D)** Representative images of isolated TA muscle fibers. Muscles were stained with α-bungarotoxin for detection of AChR (red) and antibodies directed against NF and SV2 (green) for the labeling of nerve terminals (scale bar = 20 µm). The number of AChR fragments was quantified from image stacks (control: 2 fragments, and N-EAMG: 10 fragments) **(E)** Quantitative analysis of the AChR-labeled area. **(F)** The number of AChR fragments isolated per NMJ. Each round point corresponds to one NMJ and each red square point corresponds to the mean value for each mouse. P-values were assessed using the Mann-Whitney test and indicated when p<0.05.

As MG symptoms had already reached their maximum 4 weeks after injections, we tested an alternative protocol to shorten the duration of the experiment. Mice were induced over a 4-weeks period with i.p. injections of poly(I:C)/LPS and 10 µg of T-AChR twice weekly and the persistence of symptoms analyzed 4 weeks later. At the end of the experiment, we observed that the symptoms persisted (**Figure 4A**), and that anti-T-AChR and anti-m-AChR antibodies remained elevated (**Figures 4B, C**). In addition, upon analyzing the morphology of the NMJ (**Figure 4D**), we observed a persistent decrease in the AChR area at the endplates (**Figure 4E**) and significant fragmentation of the AChR network in N-EAMG compared to control mice (**Figure 4F**). These results demonstrated the maintenance of NMJ alterations in the N-EAMG model.

These experiments showed the persistence of T-AChR and m-AChR antibodies, associated with the persistence of symptoms linked to the destruction of the NMJ, 4 weeks after an induction period of 4–6 weeks. However, symptoms appeared more severe at the end of the experiment when the induction phase lasted for six weeks, suggesting that this protocol is more effective and should be favored.

### Mechanism of sensitization in the peritoneal cavity

**Figure 5:**
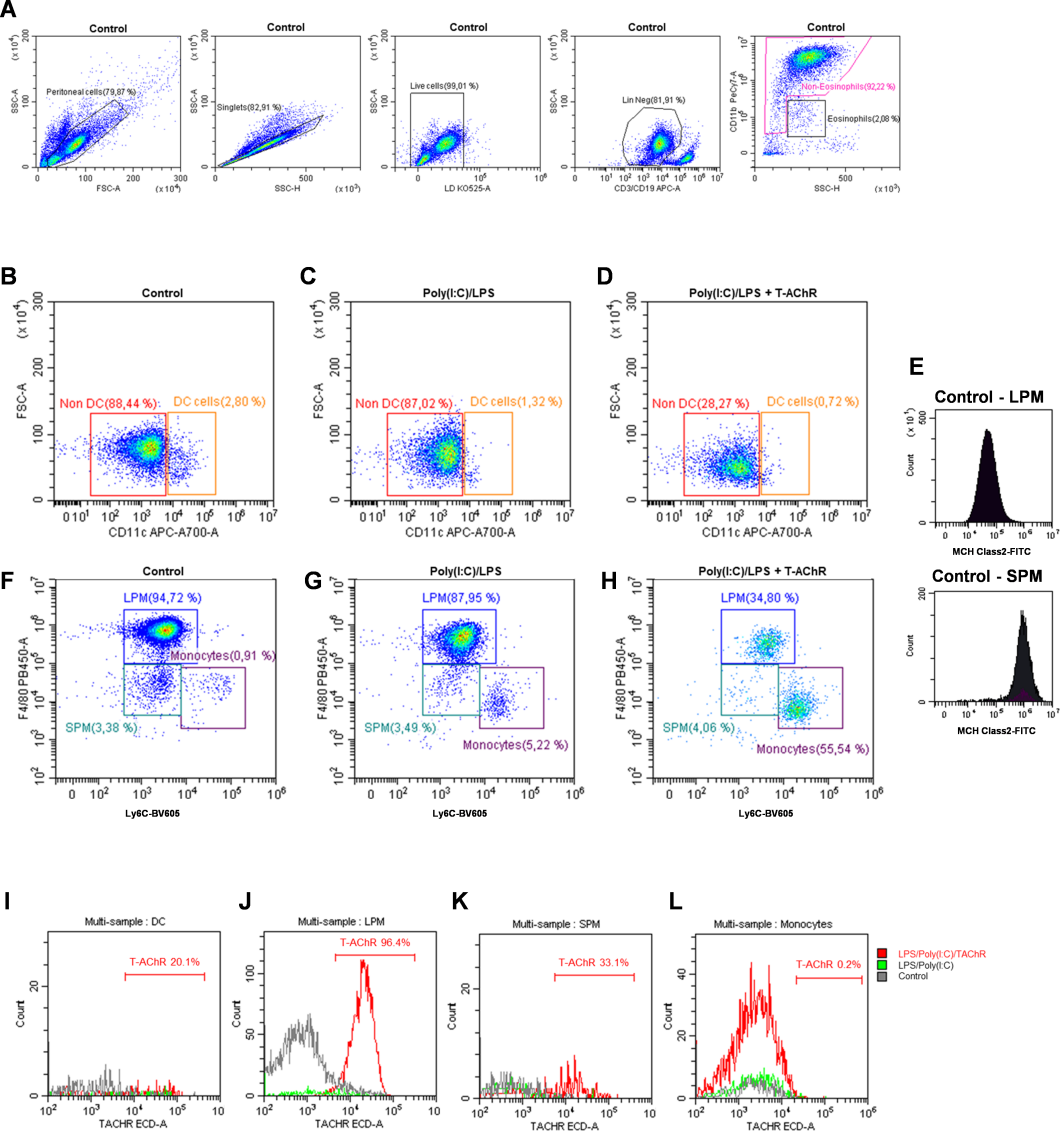
Effects of poly(I:C)/LPS and T-AChR injections on peritoneal mononuclear phagocytes. Flow cytometry analysis of peritoneal mononuclear phagocytes (representative labeling). C57BL/6 mice (n=2 per group) were i.p. injected with physiological water, poly(I:C)/LPS (PL) or poly(I:C)/LPS containing 10 µg of T-AChR-labeled with Texas-Red. Peritoneal cells were harvested after 6 h and labeled with LIVE/DEAD™ (LD34960D) and the following antibodies: anti-CD19-APC, anti-CD3-APC, anti-CD11b-PE-Cy7, anti-CD11c-AF700, anti-Ly6C-BV605, anti-F4/80-eF480, and anti-MHC Class II-FITC. **(A)** Gating strategy used to identify peritoneal, singlet, living, and lineage (CD19/CD3)-negative cells, excluding eosinophils. **(B–D)** Gating strategy for distinguishing DC from non-DC mononuclear phagocytes. **(E–H)** Monocytes, SPM, and LPM were identified based on F4/80 and Ly6C labeling and the level of MHC Class II expression **(E)**. **(I–L)** Detection of Texas-red labeled T-AChR in DC **(I)**, SPM **(J)**, LPM **(K)**, and monocytes **(L)**.

To study the mechanisms underlying sensitization to T-AChR in this new model, we analyzed mononuclear phagocytic cells in the peritoneal cavity, such as monocytes, dendritic cells (DC), and small (SPM: (CD11cneg F4/80lo LyC6int MHC2hi) and large (LPM: CD11cneg F4/80hi LyC6int MHC2lo) peritoneal macrophages. The gating strategy used for cell identification is detailed in **Figure 5**. Lineage-positive CD19^+^/CD3^+^ cells and eosinophils were excluded (**Figure 5A**). DC and non-DC mononuclear phagocytes were distinguished based on CD11c expression (**Figures 5B–D**). Finally, monocytes, SPMs, and LPMs were identified using F4/80 and Ly6C expression (**Figures 5F–H**), along with MHC Class II expression levels (**Figure 5E**). We compared the effects of LPS/poly(I:C) injections with and without T-AChR in control mice injected with physiological water. Six hours after injection, poly(I:C)/LPS (PL) induced changes in mononuclear phagocytic cells with a slight decrease in DC (**Figures 5B–D**), a decrease in LPM, and the recruitment of monocytes (**Figures 5F, G**). In the presence of T-AChR, these changes were much more pronounced in the LPM and monocytes (**Figure 5H**). Texas-red labeled T-AChR were detected primarily in the LPM and, to a lesser extent, in the SPM and DC (**Figures 5I–L**). The uptake of T-AChR by LPM, SPM, and DC was observed already 1 h after injection (data not shown).

These observations demonstrated that immunization of mice with poly(I:C)/LPS and T-AChR induced changes in peritoneal mononuclear phagocytic cells. The antigen T-AChR was taken up primarily by the LPM, but also by the SPM and DC, which could present this antigen to build an immune response against it.

## Discussion

Myasthenia Gravis (MG) associated with anti-AChR antibodies has been studied since the seventies using an experimental mouse model designated C-EAMG (2). This model is based on immunizing mice with antigens such as purified T-AChR, AChR peptides, AChR subunits, or recombinant fragments of muscle AChR emulsified in CFA (3). Over the years, the initial model has evolved little, although attempts have been made to develop alternative models. Two factors have always been mentioned as essential for successfully inducing MG symptoms in mice: the use of CFA and s.c. injections into the footpads, which lead to strong sensitization against the injected antigen (3, 4). Driven by ethical committee requirements, the C-EAMG model must be refined to avoid the use of CFA and injections into the footpads.

We develop a new EAMG model (N-EAMG) that is as efficient as the C-EAMG model. This model was obtained by immunizing mice with i.p. injections of T-AChR in a poly(I:C)/LPS adjuvant mix. As in the C-EAMG model, the N-EAMG model was characterized by the production of T-AChR, m-AChR IgG2b, and IgG1 antibodies. This new model was also affected by alterations in transmission at the NMJ, resulting from the antibody attack against AChR with a decrease in AChR surface area and an increase in AChR fragmentation. Symptoms were similar in both models but appeared more rapidly in the N-EAMG model. However, similar to the C-EAMG model, not all mice exhibited clear symptoms and the severity of the symptoms varied.

Several attempts have been made to modify the C-EAMG model. Allman et al. substituted MBT with LPS. C57BL/6 mice were immunized by several s.c. injections into the footpads and shoulders with T-AChR and LPS emulsified in IFA, compared with T-AChR emulsified in CFA. The AChR-LPS/IFA-immunized mice developed clinical signs similar to those observed following AChR/CFA immunization. They produced anti-AChR antibodies, and IgG2 and C3 deposits were detected in NMJ (6). Milani et al. described a model in which T-AChR was mixed with aluminum hydroxide for multiple s.c. injections along the back and base of the tail of C57BL/6 mice. However, these mouse models developed milder MG symptoms and lower levels of T-AChR or m-AChR antibodies. With aluminum hydroxide, mice essentially produce Th2 cytokines and anti-T-AChR IgG1, which are less pathogenic because they do not bind to the complement system (13). The induction of EAMG in mice without adjuvant was also attempted using intrathymic or intrasplenic injections combined with i.p. injections of AChR prepared from BC3H1 cells. MG symptoms and anti-AChR antibodies were observed; however, these methods were not used afterwards (14, 15). Although all of these models have shown the ability to induce antibodies against AChR and produce MG symptoms, they have not replaced the C-EAMG model. This may be because the symptoms appear less pronounced when injections are not administered into the footpads or perhaps because the use of less conventional adjuvants than CFA, which may elicit different immune responses, has hindered their adoption.

In the N-EAMG model, the main differences from the C-EAMG model are the use of a poly(I:C)/LPS adjuvant mix instead of CFA, and the use of i.p. instead of s.c. injections. How this could affect the sensitization and the development of MG symptoms is discussed below.

CFA is composed of IFA and MTB. MTB plays a central role in inducing sensitization against the injected antigen. TLR2, TLR4, TLR9, and possibly TLR8 are capable of interacting with MTB pathogen patterns. However, the activation of these pathways by CFA itself is not fully proven. Specific TLR knockout mice do not appear to be resistant to the induction of autoimmune experimental models (16). In the EAMG mouse model, CFA induces a strong Th1 response but also a Th2 response with T-AChR-specific IgG2 and IgG1 production, respectively (13, 17). LPS activates intracellular pathways through TLR4. Poly(I:C), a double-stranded RNA, is well-known to interact with TLR3 but also the protein kinase R and RNA helicases (MAD5 and RIG-I) (18). Both LPS and poly(I:C) individually induce a Th1-type immune response (19, 20). Here, we observed the induction of IgG1 and IgG2 anti-T-AChR in both the C-EAMG and N-EAMG models, suggesting that, like the C-EAMG model, the N-EAMG model also involves both Th1 and Th2 responses.

The ability of CFA or other mineral oils to induce a strong immune reaction with autoantibody production in experimental autoimmune animal models is partly due to the formation of granulomas, such as after s.c. injection into the footpads in the C-EAMG model (21) or injection in the peritoneal cavity in the pristane-induced lupus model (22). Granulomas are formed because of chronic immune responses to persistent irritants, infections, or foreign substances. They correspond to an organized aggregation of immune cells, primarily macrophages, which can phagocytose pathogens, dead cells, and other immune cells (23). Ectopic lymphoid tissues expressing chemokines and inflammatory molecules are observed in granulomas (22). Here, using i.p. injections of poly(I:C)/LPS injections in the peritoneal cavity, we did not observe granulomas. Nevertheless, we showed the recruitment of monocytes and a decrease in the proportion of LPM with poly(I:C)/LPS injections, and even more in the presence of T-AChR. The mouse peritoneal cavity contains many cell types including macrophages, DC, B cells, and T cells. In particular, there are two types of macrophages that differ in their ontogeny, phenotypic markers, and functions, including their ability to present antigens. LPM (CD11c^-^ F4/80^hi^ LyC6^int^), of embryonic origin, are primarily immunoregulatory but can acquire antigen-presenting capabilities under inflammatory conditions, whereas SPM (CD11c^-^ F4/80^lo^ LyC6^int^), derived from monocytes, are potent antigen-presenting cells with high MHC-II expression, efficiently activating T cells. In the peritoneal cavity, LPM are more predominant than SPM under steady-state conditions (24). Upon stimulation, the LPM tend to disappear, a phenomenon known as the macrophage disappearance reaction, in which they are believed to cluster on the peritoneal mesothelium and become undetectable in the peritoneal fluid. In contrast, the proportion of SPM increases, because they are derived from monocytes that rapidly infiltrate the peritoneal cavity after stimulation (24). Similar changes were observed in our experiments, particularly in the presence of the T-AChR. We did not observe an increase in SPM at 6 hours post-injections, and it seemed to mainly been described after 24 hours (25). We observed that LPM efficiently uptake T-AChR but with a low-level expression of MHC Class II molecules they might be weaker antigen-presenting cells. In contrast, SPM took up fewer TAChR molecules but expressed high levels of MHC Class II and are potent antigen-presenting cells. These results suggest that both macrophage types, albeit with varying efficiencies, can present T-AChR to immune cells locally or in draining lymph nodes, leading to a specific immune response and the development of anti-AChR antibodies. However, Takenaka et al. demonstrated that SPM, but not LPM, are capable of presenting antigens to naïve CD4+ T cells (26). In addition, DC in the peritoneal cavity, were also able to uptake TAChR. The percentages of LPM and DC were lower in the poly(I:C)/LPS plus TAChR condition. One possible explanation might be that once antigen-presenting cells are loaded with antigen, they undergo activation and upregulate chemokine receptors and adhesion molecules, which enhance their ability to migrate more rapidly to draining lymph nodes. However, we have not performed a biodistribution analysis of labeled T-AChR and antigen-sensitized cells in our model. In the C-EAMG model, mice receive s.c. injections in the footpads and at five sites on the back, leading to the rapid involvement of multiple lymph nodes, including the popliteal, inguinal, and axillary lymph nodes… In contrast, with i.p. injections, the mesenteric and mediastinal lymph nodes are the primary draining nodes of the peritoneal cavity. In both cases, molecules and immune cells carrying the antigen will eventually reach the spleen (27). An immune response may differ depending on the localization of the lymph nodes and the distinct tissues they drain, which are exposed to different antigens. Lymph nodes can thus contain specialized immune cell populations (28). This might explain that the sensitization is more rapid in the N-EAMG model.

In C57Bl/6 mice, AChR-MG obtained with i.p. injections of poly(I:C) alone or with poly(I:C) and LPS is associated with thymic changes due to the expression of interferon type I. This leads to the overexpression of α-AChR subunit (the primary immunogenic subunit), the overexpression of CXCL13, CCL21, and BAFF which can induce B-cell recruitment and proliferation (7, 9). However, these thymic changes tend to rapidly disappear over time. Nevertheless, we demonstrated that after 6 weeks of i.p. injections of poly(I:C) and LPS, some thymic changes were maintained (9). In one experiment, we analyzed B cells in the thymus 6 weeks after i.p. injections of poly(I:C)/LPS and T-AChR. A slight but non-significant increase in the number of B cells was observed by immunofluorescence on thymic sections, along with a modest increase in CD19 mRNA expression (data not shown), similar to the findings of Robinet et al., but with less supporting evidence. In this N-EAMG model, we did not achieve lymphofollicular hyperplasia similar to human disease. It is possible that C57Bl/6 mice are more resistant than other mouse strains. Recently, Pinto et al. demonstrated the accumulation of thymic B cells and the putative development of ectopic germinal centers in non-obese diabetic (NOD) aging mice, but not in C57Bl/6 mice (29). In addition, Hidalgo et al. observed an increase in the number of thymic B cells and germinal center-like structures in BWF1 mice, a murine model of systemic lupus erythematosus (SLE), from 5 weeks of age (30). In the 1970s, the susceptibility to EAMG of different strains with various H-2 haplotypes was explored. C57Bl/6 mice with the H-2b haplotype were among the most susceptible to EAMG and subsequently became the preferred model in all studies (2, 3). Here, we did not explore the influence of genetic background on N-EAMG susceptibility but it could be of interest to explore if this might favor the development of MG-associated thymic changes.

The inflammatory status of the thymus is also a key element in B cell recruitment (31). Hodge et al. showed that the Th1 inflammatory/infectious process favors the recruitment of peripheral T and B cells. This effect is dependent on, but not exclusive to, the loss of thymus cellularity and available space in the thymus, combined with the expression of chemokines and cytokines (32). Therefore, in addition to the genetic background of the mice, factors such as the sanitary status of the animal facility and the age of the mice could influence the recruitment of thymic B cells.

In conclusion, we have demonstrated that the N-EAMG model is as efficient as the C-EAMG model. In addition, it overcomes several limitations of the C-EAMG model: symptoms appear more rapidly, i.p. injections are simpler for experimenters and less traumatic for mice. This model also prevents footpad inflammation, which can alter clinical measurements due to swelling. To further support the efficacy of this new immunization protocol, it should be tested in the MuSK-MG model in collaboration with specialists. To confirm the efficacy of this model, it will also be crucial to test it as a preclinical model in parallel with the C-EAMG model, with the hope that it can eventually replace it in the long term.

## Data Availability

The raw data supporting the conclusions of this article will be made available by the authors, without undue reservation.
