# Supplementary material for: Development of a refined experimental mouse model of myasthenia gravis with anti-acetylcholine receptor antibodies
Source: Front Immunol. 2025 Mar 31;16:1521382. doi: 10.3389/fimmu.2025.1521382 (PMC11994731; doi:10.3389/fimmu.2025.1521382)
Supplement: Supplementary file 1 [file DataSheet1.docx]

## Supplemental Figure 1: Establishment of a Global Clinical Score


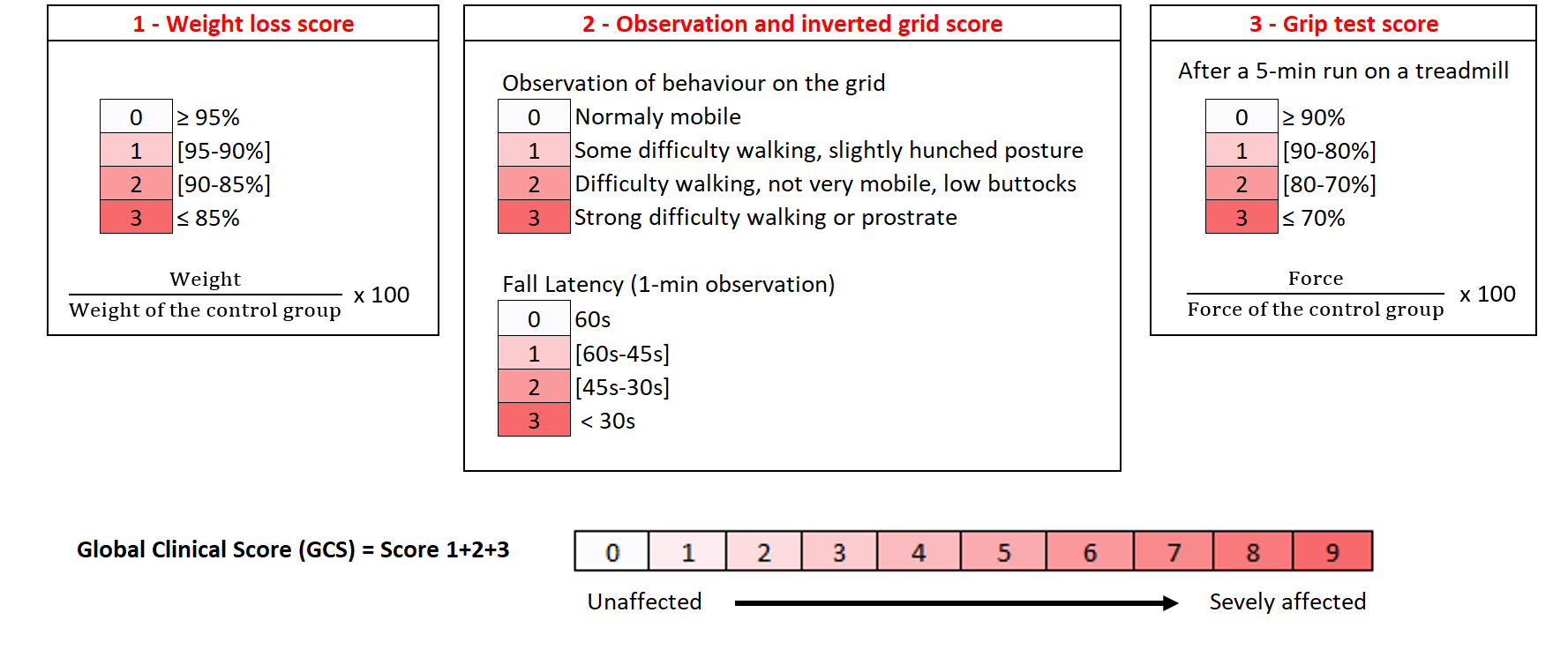


Different assessments were performed to evaluate the clinical state and to calculate a global clinical score for each animal. (1) Mice were weighed weekly and given a score of 0-3. (2) An inverted grid test was performed by gently dragging the mouse across the top grid of the cage 10 times. The grid was then rotated, and the time at which each mouse fell off was recorded. During all tests, the behaviors of the mice, such as posture and gait, were also recorded. A score from 0 to 3 is given, taking into account separately the grid test and the observation. (3) Muscle strength was analyzed by measuring forelimb strength using a grip strength apparatus (Bio-GS3, Bioseb, France) after a 5-min run on a treadmill (14cm/s) and given a score of 0-3. A global clinical score ranging 0–9 was then calculated. The mice were considered sick when they reached a global clinical score of 2. Mice with a global clinical score of 9 were euthanized.

## Supplemental Figure 2: Comparison of the N-EAMG and C-EAMG models for MG symptoms - Detailed clinical parameters


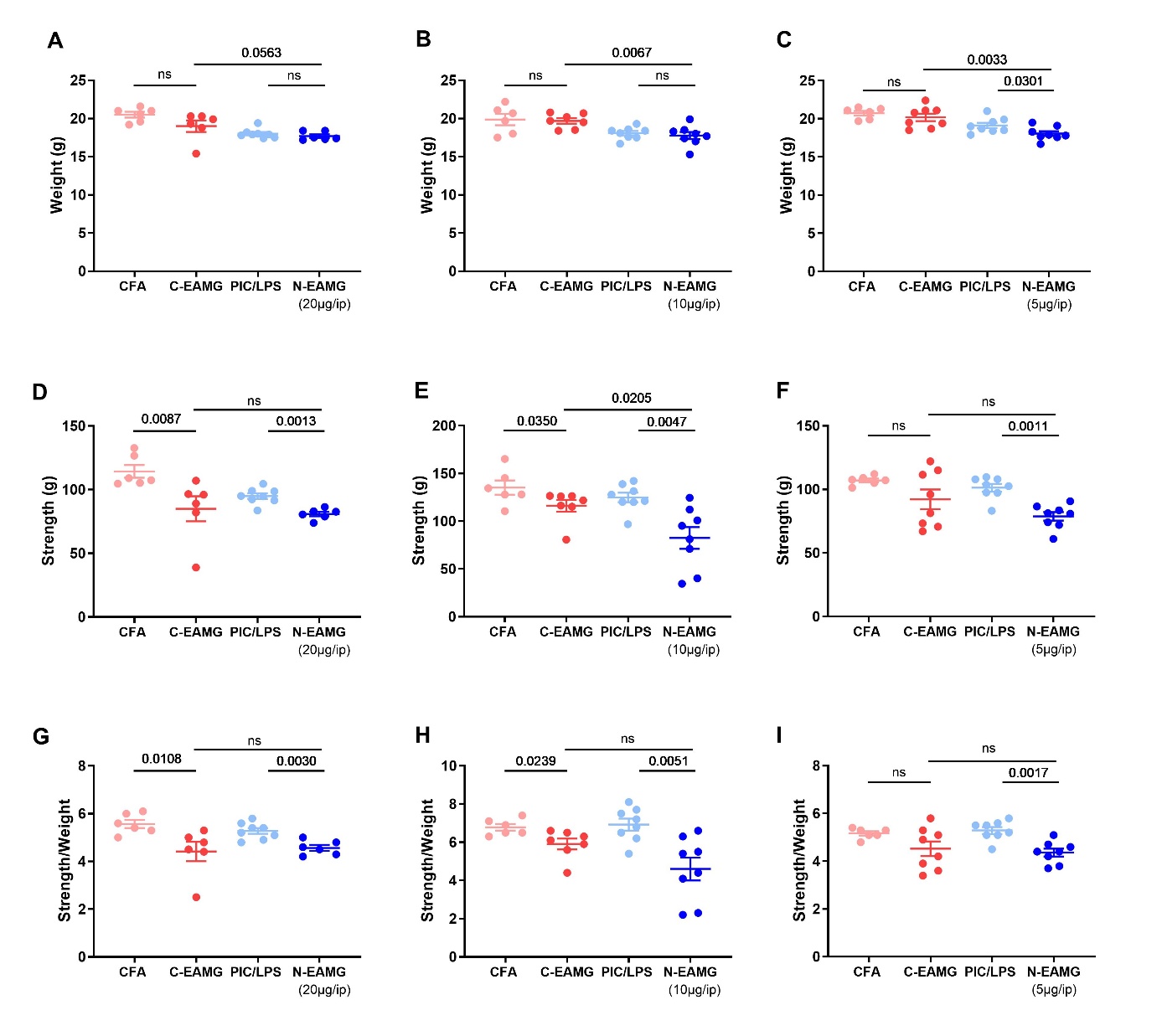


C57BL/6 mice (n=6–8 per group) were used in three different experiments with different doses of T-AChR for the N-EAMG model. Mice were i.p. injected twice weekly with a poly(I:C)/LPS (PL) adjuvant mix or a PL adjuvant mix with T-AChR (N-EAMG) 20 µg (A, D, G), 10 µg (B, E, H), or 5 µg (C, F, I). For the C-EAMG model, mice were immunized with CFA/T-AChR (30 µg, C-EAMG) or just CFA on day 0 and boosted after 3–4 weeks. Clinical evaluations were performed after 6 weeks by measuring weight loss (A–C), strength (D–F), and ratio strength/weight (G–I). P-values were assessed using the Mann-Whitney test to compared CFA and C-EAMG, PL and N-EAMG, C-EAMG and N-EAMG, and indicated when p<0.1.

## Supplemental Figure 3: Comparison of the N-EAMG and C-EAMG models for MG symptoms – measure of anti-T-AChR and anti-m-AChR antibodies


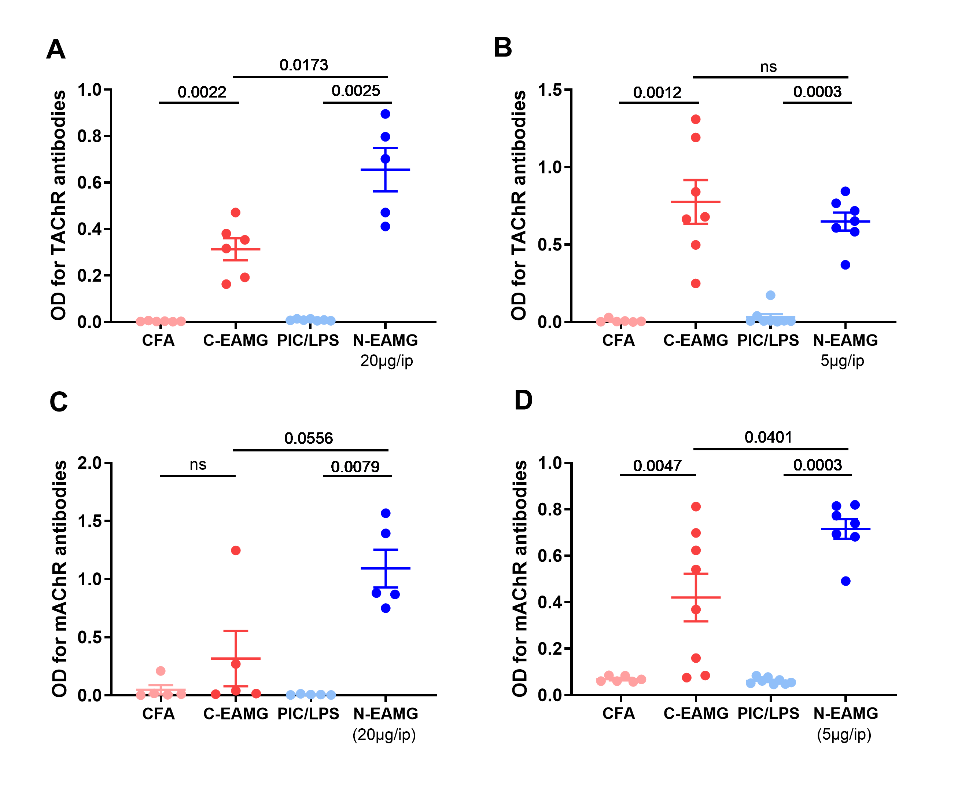


C57BL/6 mice (n=6–8 per group) were used in different experiments with different doses of T-AChR for the N-EAMG model. Mice were i.p. injected twice weekly with a poly(I:C)/LPS (PL) adjuvant mix or a PL adjuvant mix with T-AChR (N-EAMG) 20 µg (A, C) or 5 µg (B-D). For the C-EAMG model, mice were immunized with CFA/T-AChR (30 µg, C-EAMG) or just CFA on day 0 and boosted after 3–4 weeks. (A–B) Anti-T-AChR and (C–D) anti-m-AChR antibodies were measured using ELISA and detected using anti-mouse IgG antibody. P-values were assessed using the Mann-Whitney test to compared CFA and C-EAMG, PL and N-EAMG, C-EAMG and N-EAMG, and indicated when p<0.1.
